# MHC Class II and Beyond: Complex Role of CD74 in Cancer

**DOI:** 10.3390/cells15020128

**Published:** 2026-01-11

**Authors:** Joanna Bandola-Simon, Paul A. Roche

**Affiliations:** Experimental Immunology Branch, National Cancer Institute, National Institutes of Health, Bethesda, MD 20892, USA; joanna.bandola@nih.gov

**Keywords:** invariant chain, CD74, MHC class II, MIF, antigen presentation, cancer, immunotherapy

## Abstract

**Highlights:**

**What are the main findings?**

- CD74 links MHC class II antigen presentation with signaling and transcriptional regulation in cancer.
- Dysregulated CD74 processing and MIF-CD74 signaling contribute to tumor immune evasion.

**What are the implication of the main findings?**

- CD74 shapes the tumor immune microenvironment and antitumor immune responses.
- CD74 represents a biomarker and therapeutic target for cancer immunotherapy.

**Abstract:**

Invariant chain, also known as CD74 when expressed on the plasma membrane, is classically recognized for its role in Major Histocompatibility Complex class II molecule assembly, trafficking, and peptide loading in professional antigen presenting cells. However, recent studies implicate CD74 as a broader regulator of tumor–immune interactions, modulating antigen presentation, cytokine signaling, and immune evasion across diverse cancers. This review synthesizes emerging evidence that CD74 functions as a “master regulator” of antigen presentation in cancer, integrating its canonical chaperone role with its noncanonical role in transcription regulation and in signaling via macrophage migration inhibitory factor. We explore how tumor microenvironmental contexts redefine CD74 biology, influencing antitumor immunity and therapeutic outcomes.

## 1. Introduction

Antigen presentation is central to both acquired and adaptive immune responses. Effective antitumor immunity depends on the presentation of tumor-derived peptides that T cells can only recognize when presented in the context of Major Histocompatibility Complex (MHC) molecules [1]. While broadly expressed MHC class I (MHC-I) molecules are involved in priming cytotoxic CD8^+^ T cells, MHC class II (MHC-II) molecules (whose expression is restricted primarily to professional antigen presenting cells (APCs)) play a crucial role in shaping CD4^+^ T helper responses, therefore indirectly orchestrating cytotoxic T cell responses. An essential player in MHC-II pathway is the chaperone molecule called Invariant chain (Ii), also known as CD74. Primarily known for its role in antigen presentation, Ii plays a critical role in adaptive immunity and therefore also in pathologies such as cancer.

Beyond its known role as a chaperone for MHC-II molecules, Ii has emerged as a multifunctional protein with relevance far beyond antigen presentation. Firstly, Ii ensures proper folding and transport of MHC-II molecules to endosomal/lysosomal antigen processing compartments for peptide loading, thereby enabling mature peptide–MHC-II (pMHC-II) complexes to reach the cell surface [2]. This tightly regulated process is essential for effective activation of CD4^+^ T cells and helps shape the quality and magnitude of antitumor immune responses.

Secondly, in recent years, CD74 has gained attention as a signaling molecule, particularly through its function as a high-affinity receptor for macrophage migration inhibitory factor (MIF) [3]. Engagement of CD74 by MIF initiates a signaling cascade that ultimately promotes cell survival and proliferation. This signaling axis has been implicated in chronic inflammation, autoimmunity, and in the tumor microenvironment (TME), where it can support protumorigenic processes. Elevated expression of CD74 has been observed in a wide range of malignancies, highlighting its potential role in tumor biology beyond antigen presentation. Moreover, CD74 expression in cancer cells and tumor-associated immune cells is increasingly recognized as a double-edged sword. This dual functionality has made CD74 an attractive target for immunotherapy.

Given these diverse functions, CD74/Ii represents a critical intersection point between tumor cell biology, antigen presentation, and immune regulation. Understanding how CD74 expression, processing, and signaling are altered in cancer is essential for deciphering its contribution to tumor progression and for optimizing strategies to therapeutically exploit this molecule.

## 2. Canonical Role of Ii in Antigen Presentation

Activation of antigen-specific CD4^+^ T cells relies on their loading and presentation on MHC-II molecules, expressed on APCs. For efficient antigen presentation, MHC-II molecules must traffic intracellularly to endosomal antigen processing compartments, where they bind internalized and degraded fragments of foreign antigens (reviewed in [2]). The trafficking of newly synthesized MHC-II is directed by its association in the endoplasmic reticulum (ER) with the chaperone protein Ii that is crucial for proper folding and transport within the cell [4].

### 2.1. MHC-II Chaperone

Ii is a non-polymorphic (hence invariant) type II transmembrane glycoprotein. The primary sequence of Ii genes is highly conserved between species. Resulting from alternative splicing and use of alternative translation start sites, there are four isoforms of Ii in humans (the long isoforms p43 and p41, and the short isoforms p35 and p33) and two Ii isoforms in mice (p41 and p31) [5]. The human p35 and p43 isoforms contain an arginine-based retention motif within their cytosolic N-terminal extension, which confines them to the ER unless they are in complex with MHC-II molecules, in which case this retention motif is masked [6]. In contrast, isoforms p33 and p41 lack this regulatory feature and are therefore capable of trafficking to post-ER compartments, even in cells that do not express MHC-II [7].

Since the transcription of both MHC-II and (to some extent) Ii is regulated by the MHC class II transcriptional activator (CIITA), most cells expressing Ii also express MHC-II molecules. In the context of MHC-II biology, Ii plays several essential roles: it assists in the proper folding of MHC-II, it guides MHC-II-Ii complexes to the appropriate endosomal compartments, and crucially, it occupies the peptide-binding groove of MHC-II to prevent premature peptide loading [2]. During MHC-II biosynthesis in the ER, Ii assembles with MHC-II α and β chains to form a nonameric complex (an Ii trimer with three MHC-II heterodimers) which leaves the ER and traffics to antigen processing compartments [8,9].

### 2.2. Ii Processing and Peptide Exchange

To enable binding and presentation of exogenous peptides by MHC-II complexes, Ii undergoes sequential proteolytic cleavage in endosomal/lysosomal antigen processing compartments. To prevent premature binding of other peptides, the peptide-binding groove of MHC-II is occupied by a specific segment of Ii called CLIP (class II-associated Ii peptide), which must be displaced before antigenic peptides can bind [10,11,12]. CLIP is generated in a series of proteolytic steps: the full length 33–35 kDa Ii protein is initially cleaved to produce a 22–25 kDa intermediate (Iip22), which is subsequently processed to form Iip10, followed by its cleavage to CLIP [13]. The proteolytic cleavage to generate CLIP is catalyzed by specific enzymes: cathepsin S in B cells, macrophages and DCs, and by cathepsin L in thymic epithelial cells [14,15,16]. The MHC-II-CLIP complex is then targeted by non-classical MHC-II molecules (HLA-DM in humans/H2-M in mice and HLA-DO in humans/H2-O in mice), which catalyze the exchange of CLIP for antigenic peptides [17,18,19].

The kinetics of Ii degradation by cathepsins also modulates DC motility by inducing a discontinuous cell migration pattern with alternating high- and low-motility phases. This regulation depends on the actin-based motor protein myosin II, through its association with Ii [20], showing yet again a multifaceted role of Ii in cell biology.

### 2.3. Regulation of Ii by CIITA and IFNγ

Transcription of genes encoding conventional MHC-II molecules and the MHC-II accessory proteins HLA-DM and HLA-DO require expression of CIITA. In addition to professional APCs, CD74 expression is detectable in various non-hematopoietic cell types, including endothelial, epithelial, and certain mesenchymal cells (as well as malignant cells), and its expression in these cells is markedly upregulated by specific stimuli such as IFN-γ [21]. CIITA is required for interferon (IFN)-γ-induced expression of MHC-II and (to some extent) of CD74, and in fact CIITA itself can be induced by IFN-γ [22]. Tumor suppression of this axis results in immune evasion [23], thereby linking MHC-II and CD74 expression to cancer. CD74 expression does not absolutely require CIITA in resting spleen APCs [24], demonstrating that CD74 is regulated by both CIITA-dependent and CIITA-independent transcriptional pathways [24,25]. The fact that expression of MHC-II is strictly dependent on CIITA, whereas expression of CD74 is not, suggests a functional role for CD74 that is independent of MHC-II, a point that will be addressed below.

## 3. Beyond Chaperoning: CD74 as a Signaling Hub

Although the role of Ii in MHC-II assembly and targeting has been well characterized, its specific functions at the cell surface have not been completely elucidated. It has long been established that newly synthesized CD74 briefly appears at the cell surface before undergoing rapid internalization into the endosomal pathway. Studies examining surface-expressed CD74 are challenging, as the molecule is only transiently expressed on the cell surface. CD74 displays a remarkably short surface half-life, estimated at less than 10 min. Owing to this rapid internalization and continuous replenishment by newly synthesized molecules, approximately 4 × 10^6^ CD74 molecules are cycled to the cell surface per cell per day in B cell lymphoblastoid cell line. Interestingly, 2–5% of Ii is expressed on the surface independent of MHC [26,27,28], additionally pointing to a potential role of CD74 beyond its MHC-II chaperone function.

### 3.1. CD74 as a Cytokine Receptor

Originally characterized as a T cell-derived inhibitor of macrophage motility [29,30], MIF is now recognized as a ubiquitously expressed cytokine, present in most solid and hematologic malignancies. While classically defined as a pro-inflammatory mediator of innate immunity, accumulating evidence indicates that, within TME, MIF drives an anti-inflammatory, immune-evasive, tolerogenic program across innate and adaptive immune cells (reviewed in [31]). The role of MIF in monocyte/macrophage biology was thoroughly studied since its discovery in the 1960s; however, it was not until 2003 that CD74 was reported as a high-affinity receptor for MIF [3].

### 3.2. Mechanism of MIF-CD74 Action

Although CD74 is traditionally known as the chaperone of MHC-II, its function as a high-affinity receptor for MIF has revealed a distinct signaling role for this molecule. CD74 lacks a substantial cytoplasmic signaling domain, possessing only a short intracellular tail insufficient to initiate downstream phosphorylation events on its own. Consequently, MIF binding to CD74 is necessary (but not sufficient) to trigger intracellular signaling, and the discovery of essential co-receptors has been central to understanding this pathway. CD44 was identified as the first co-receptor required for CD74-dependent signaling, forming a complex that enables recruitment of downstream kinases [32]. Subsequently, chemokine receptors such as CXCR2 and CXCR4 have been reported to partner with CD74 in certain cell types, broadening the repertoire of MIF-responsive signaling modules [33,34].

Upon MIF engagement, the CD74-CD44 complex initiates several important signaling cascades involving the activation of Src kinase and subsequent phosphorylation of ERK1/2 MAP kinases [35]. This complex also stimulates the PI3K/Akt pathway, known to play a crucial role in production of cytokines such as prostaglandin E2, in cell survival [3,36] and, importantly, in the development of cancer [37]. Beyond the membrane-proximal MIF signaling events, CD74 also participates in a second layer of signaling through regulated intramembrane proteolysis (RIP). Following engagement and internalization, CD74 undergoes sequential cleavage by cathepsins and intramembrane proteases such as SPPL2a and SPPL2b [38,39]. This process releases the cytoplasmic CD74 intracellular domain (CD74-ICD), which translocates to the nucleus and triggers NF-κB-dependent transcription, regulating genes involved in cell maturation, survival, and immune activation [40,41,42].

**Figure 1:**
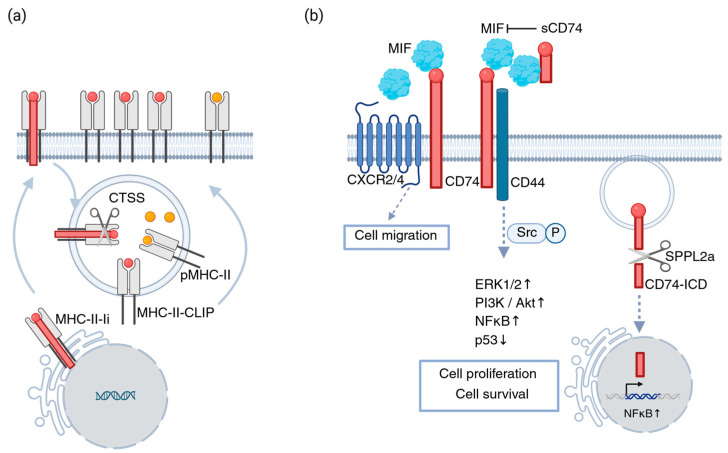
Multifaceted roles of CD74 in cancer. (**a**) CD74/Ii chaperones MHC-II molecules from the ER to endo/lysosomal compartments, where Ii is degraded to CLIP, which is then exchanged for antigenic peptides for CD4^+^ T cell presentation; tumors can suppress this process by increasing CLIP retention; (**b**) CD74 also functions as a receptor for MIF, forming a complex with CD44 to activate cell survival and proliferation pathways. A soluble form of CD74 can inhibit MIF-CD74 signaling and induce apoptosis. SPPL2a-mediated cleavage releases the CD74 intracellular domain (CD74-ICD), further promoting cell survival signaling. CD74 can also associate with CXCR2/4 to induce migration-related pathways. Created in BioRender. Bandola-Simon, J. (2026) https://BioRender.com/bg3ba5g (accessed on 5 January 2026).

Altogether, these pathways (summarized in Figure 1, Bandola-Simon, J. (2026) https://BioRender.com/bg3ba5g (accessed on 5 January 2026)) highlight CD74 as a multifunctional signaling hub integrating extracellular cues from MIF with intracellular activation programs. In the context of cancer, dysregulation of these mechanisms can shift CD74′s role toward protumorigenic signaling, underscoring its importance as a therapeutic target, which we discuss in the next chapters.

### 3.3. CD74 in B Cell Development and Survival

CD74 plays an essential role in B cell biology that extends beyond its well-known function as a chaperone for MHC-II. Evidence from CD74-deficient mice revealed profound defects in B cell maturation, including reduced numbers of mature follicular B cells and impaired germinal center development. Importantly, many of these defects persist even when MHC-II function is rescued, demonstrating that CD74 has intrinsic signaling activities in B cells that are independent of MHC-II-related antigen presentation activity [43]. This MHC-II-independent role of CD74 is connected to intramembrane cleavage of Ii by SPPL2a protease, thereby generating a cytoplasmic CD74-ICD. CD74-ICD translocates to the nucleus and functions as a transcriptional regulator that enhances the expression of genes critical for B cell survival, most notably NF-κB p65/RelA-dependent survival genes such as Bcl-2, Bcl-xL, as well as regulators of metabolism and cell growth [38,39]. The physiological significance of this pathway is highlighted in SPPL2a-deficient mice, which phenocopy many aspects of CD74 deficiency. Loss of SPPL2a leads to the accumulation of uncleaved CD74 that impairs vesicular trafficking and homeostasis in late endosomes and lysosomes. This results in a developmental block at the transitional B cell stage and leads to a dramatic reduction in mature B cells. The striking similarity between SPPL2a-deficient and CD74-deficient B cell phenotypes demonstrates that intramembrane cleavage of CD74 and the release of CD74-ICD is required for normal B cell maturation, rather than mere degradation of CD74 [44,45]. As a result, CD74-ICD-dependent signaling is critical not only during transitional stages but also for maintaining homeostatic survival of peripheral B cell pools [46]. The role of CD74 as a regulator of transcription in normal B cells has been recently shown to be impaired in malignant B cells, where CD74-ICD no longer interacts with transcription factor PAX5 that regulates the tumor suppressor gene DMTF1 [47].

Overall, these findings position CD74 as a dual-function molecule in B cells: a classical MHC-II chaperone and a signaling receptor whose intramembrane proteolysis generates a transcriptionally active ICD fragment, indispensable for B cell maturation, differentiation, and survival, as well as protection from malignant transformation, as described in the following chapter.

## 4. CD74 Expression in Cancer

Beyond its role in adaptive immunity as a chaperone for MHC-II molecules during antigen processing and presentation to CD4^+^ T cells in steady-state, CD74 has emerged as a signaling receptor with key implications in cancer biology. The discovery that CD74 functions as a high-affinity receptor for MIF revealed a new signaling axis that directly contributes to oncogenesis. Binding of MIF to CD74 initiates the intracellular signaling cascades that promote cell proliferation, survival, and resistance to apoptosis hallmarks of malignant transformation that tumor cells readily exploit.

The relevance of immune surveillance in controlling malignancy is underscored by the observation that transplant patients receiving immunosuppressive therapy rarely develop solid tumors but are disproportionately affected by hematologic malignancies [48,49]. This highlights the unique vulnerability of hematologic tissues to disruptions in immune regulation and antigen presentation, processes in which CD74 is deeply involved. On one hand, high expression of CD74 in cancer may enhance antigen presentation capacity and promote antitumor immune activation. On the other hand, tumor-intrinsic MIF-CD74 signaling can drive oncogenic pathways, support immune evasion, and promote an immunosuppressive microenvironment.

### 4.1. CD74 Expression Patterns and Its Functional Duality in Cancer

Under physiological conditions, CD74 expression is largely restricted to professional APCs, including B cells, DCs, and monocytes/macrophages. In cancer, however, CD74 expression becomes aberrantly widespread. Tumor-intrinsic CD74 has been documented in a broad spectrum of malignancies (as summarized in Table 1).

In many types of cancer, overexpression of CD74 is accompanied by enhanced MIF-CD74 signaling, which drives tumor progression, metastasis, and poor clinical outcomes. Conversely, in certain contexts, particularly where CD74 co-expression with MHC-II supports antigen presentation, high CD74 expression may correlate with improved survival [50], likely reflecting increased immune visibility of tumor cells. This dual role illustrates the complex, context-dependent nature of CD74′s contribution to cancer biology.

Mechanistically, tumor cells can express distinct CD74 isoforms that differ in intracellular trafficking and MHC-II interactions. Post-translational modifications, including glycosylation and phosphorylation, further refine CD74′s localization and function [51,52,53]. While CD74 transcription is inducible by inflammatory cytokines such as IFN-γ and TNF-α, oncogenic pathways can also sustain its expression independently of CIITA, revealing multiple regulatory layers that integrate immune and oncogenic signals.

**Table 1:** CD74 expression in different types of cancer.

Cancer Type	Reported Finding	References
B cell lymphomas	MHC-II-CLIP increase, HLA-DM loss	[54,55,56,57]
Multiple Myeloma	CD74 overexpression, therapy target	[55]
Glioma	CD74 overexpression	[58]
B cell Leukemia	MHC-II-CLIP increase	[59]
PDAC	CD74 overexpression	[60]
Hepatocellular carcinoma	CD74 overexpression	[61]
Breast cancer	CD74/MIF overexpression	[62]
Melanoma	CD74/MIF, serum sCD74 increase	[56,63,64,65,66]
Bladder cancer	CD74 expression, therapy target	[67]
Gastric cancer	CD74 overexpression, prognostic potential	[68]
Renal cancer	CD74 overexpression, prognostic potential	[69]
Lung cancer	CD74 overexpression, prognostic potential	[70]
Sarcoma	CD74 overexpression, biomarker	[71]
Thymic epithelial neoplasms	CD74 overexpression, biomarker	[72]
Cervical cancer	Therapy target	[73]
*Other reports*		
Tumor-infiltrating Tregs	CD74 overexpression	[74]
Tumor-draining lymph node cDC2	MHC-II-CLIP increase, H2-DM loss	[75]

#### 4.1.1. CD74 as a Signaling Hub in the TME

The MIF-CD74 axis plays divergent role in immune versus tumor cells. In macrophages and DCs, it enhances cytokine secretion and antigen presentation, amplifying antitumor immune activation. By contrast, in tumor cells, the same signaling promotes immune evasion, proliferation, and survival, contributing to the formation of an immunosuppressive microenvironment. Thus, CD74 acts as a molecular switch that can either support or suppress immunity depending on the cellular context. In the tumor setting, persistent MIF-CD74-CD44 signaling enhances resistance to apoptosis and favors chronic inflammation, thereby reinforcing tumor progression [3,76].

Interestingly, recent studies have reported CD74 overexpression in human tumor-infiltrating regulatory T cells (Tregs) [74], suggesting a possible role in modulating the immunosuppressive compartment of the TME. This emerging evidence points to a broader immunoregulatory network orchestrated through CD74 beyond traditional antigen presenting pathways.

#### 4.1.2. CD74 and the Antigen Presentation Machinery in Lymphomas

Among hematologic malignancies, B cell lymphomas exhibit particularly strong CD74 expression and dependency. Several lymphoma subtypes exploit CD74-related mechanisms to evade immune surveillance. For example, B cell lymphomas carrying IRF8 mutations can circumvent immune detection via CD74-dependent pathways [54].

Classical Hodgkin lymphoma, as well as a subset of Hodgkin’s Disease, provide notable cases of disrupted MHC-II antigen presentation. Enhanced level of MHC-II occupied by CLIP molecules, rather than antigenic peptides, were reported in patient samples for both of these hematological disorders, with the loss of HLA-DM expression coinciding with increased MHC-II-CLIP in classical Hodgkin lymphoma [55,57].

#### 4.1.3. CD74 Expression in Solid Tumors

Beyond lymphoid malignancies, CD74 expression has been documented in a diverse array of solid tumors (Table 1), including bladder [67], gastric [68], renal [69], non-small cell lung cancer [70], thymic epithelial neoplasms [72], and certain sarcomas [71]. In these types of cancer, CD74 frequently co-localizes with MIF at the tumor-stromal interface, activating downstream signaling pathways that sustain tumor growth and metastasis. The extent to which CD74 contributes to immune modulation in solid tumors appears to depend on both the cellular source (tumor vs. stromal) and the dominant signaling environment [31]. In some cancers, CD74 expression aligns with an inflamed tumor phenotype and higher immune infiltration, whereas in others it marks aggressive, immune-resistant disease.

MIF contributes to breast cancer development by enhancing tumor cell proliferation, metastasis, and blood vessel formation, while also shaping the TME (reviewed in [62]). Elevated MIF expression in breast cancer tissues and cells is correlated with poorer patient outcomes, especially in cases of triple-negative breast cancer. MIF mediates its tumor-promoting effects through binding to CD74, thereby activating pathways such as PI3K/Akt, and stimulating angiogenesis via molecules such as VEGF and IL-8. Collectively, these insights highlight MIF and its associated signaling pathways as potential therapeutic targets in cancer treatment.

#### 4.1.4. CD74 in Melanoma

In one melanoma study, IFNγ-induced CD74 overexpression and MIF-CD74 signaling was shown to increase phosphorylated AKT levels, leading to elevated inflammatory cytokine production and promoting disease progression [63]. In contrast, high tumor CD74 expression was the strongest favorable prognostic marker among several inflammatory proteins examined in advanced melanoma in other studies [56,64]. 

Although the presence of soluble CD74 (sCD74) in human plasma was first detected almost thirty years ago [77], its biological role and clinical significance have only begun to receive attention. Circulating sCD74 was first shown to play an important role in autoimmune liver disease [78]. In serum from patients with advanced melanoma, levels of sCD74 are elevated, and a high sCD74/MIF ratio in those patients predicts longer survival. sCD74, mainly the 25-kDa form derived from 33 kDa CD74 isoform, is secreted by melanoma cells and macrophages and is enhanced by IFN-γ [65]. Both recombinant and macrophage-derived sCD74 suppressed melanoma growth and triggered apoptosis by inhibiting the MIF/CD74/AKT survival pathway. Thus, sCD74-MIF interactions regulate melanoma progression and patient outcomes. These results suggest that CD74 has complex, context-dependent roles in melanoma, exhibiting both tumor-promoting and tumor-suppressive functions.

Aberrant expression of CD74 in both hematologic and solid tumors reflects its adaptability to oncogenic pressures and its role in shaping the immune landscape of cancer. These findings establish CD74 as a pivotal node at the interface between tumor cell survival and immune regulation, offering both a biomarker of immune dysfunction and a potential therapeutic target for modulating tumor immunity.

### 4.2. Ii Processing in Cancer: Cathepsins and CLIP

As described in detail in Chapter 1, Ii plays a pivotal role in the MHC-II antigen presentation pathway by stabilizing newly synthesized MHC-II molecules and guiding their trafficking through the endocytic network. During this process, Ii is sequentially degraded, leaving behind the “placeholder” CLIP peptide, which occupies the peptide-binding groove of MHC-II, until it is replaced by antigenic peptides. The exchange of CLIP for high-affinity peptides typically occurs in late endosomal or lysosomal compartments, preceding the transport of peptide-loaded MHC-II to the cell surface. As a result, CLIP-loaded MHC-II αβ complexes are largely confined to intracellular compartments of the endocytic pathway. Nevertheless, a proportion of MHC-II-CLIP complexes reach the plasma membrane, reflecting incomplete or inefficient peptide exchange [79,80].

#### 4.2.1. Enhanced MHC-II-CLIP Expression and Tumor Growth

Accumulation of MHC-II-CLIP complexes has been implicated in immune evasion within TME. In hematological malignancies such as acute myeloid leukemia, the presence of CLIP on leukemic blasts was reported to correlate negatively with patient survival [59]. Reduced surface CLIP expression was associated with increased activation and polarization of Th1 CD4^+^ T cells, suggesting that efficient removal of CLIP is critical for mounting effective anti-leukemia immune responses [59].

Direct evidence linking aberrant CLIP retention to tumor progression was demonstrated by our lab in a transplantable mouse tumor model. We showed that conventional type 2 DCs (cDC2s) in tumor-draining lymph nodes display elevated levels of MHC-II-CLIP complexes and a concomitant reduction in the ability of these MHC-II molecules to load exogenous antigenic peptides. Consequently, these cDC2s fail to effectively prime CD4^+^ T cells, thereby skewing T cell responses towards a Th2 phenotype and leading to impaired antitumor immunity [75]. By generating mice possessing a mutation in Ii that enhances CLIP affinity for MHC-II, we found that tumors transplanted subcutaneously or administered intravenously grew significantly faster in Ii-mutant mice than in wild-type mice, highlighting a functional link between inefficient CLIP removal and enhanced tumor progression. A similar finding was reported in Hodgkin lymphoma and, as in our mouse studies, dysregulated expression of H2-M was thought to be responsible for increased MHC-II-CLIP expression [55,57]. Tumor-mediated accumulation of MHC-II-CLIP therefore represents yet another strategy by which tumors subvert the MHC-II antigen presentation pathway to escape immune recognition.

#### 4.2.2. Cathepsins and Cancer

Proteolytic degradation of Ii is mediated by a family of lysosomal cysteine proteases known as cathepsins, among which cathepsin S (CTSS) plays a particularly central role in DCs and B cells. CTSS cleaves Ii to generate CLIP and facilitates subsequent peptide exchange in the MHC-II groove by H2-M. Loss (or inhibition) of CTSS results in the accumulation of Ii degradation intermediates that cannot be removed by H2-M, thereby impairing antigen presentation to CD4^+^ T cells [16].

CTSS has been widely studied for its extracellular roles in the TME, contributing to matrix remodeling and angiogenesis in solid tumors [81,82,83]. However, its intracellular activity in immune cells in cancer has only recently been investigated. Our work demonstrated that CTSS activity is upregulated specifically in cDC2s in tumor-draining lymph nodes, contributing to CLIP accumulation and linking enhanced protease function to altered antigen presentation in antitumor responses [75]. Dysregulation of CTSS activity was also recently reported for hematological malignancies: CTSS mutations, such as the recurrent Y132D hotspot in follicular lymphoma, enhance enzymatic activity and modulate antigen processing, influencing both CD4^+^ and CD8^+^ T cell-mediated responses [84]. This study also found that inhibition of CTSS diversifies the repertoire of presented antigens, thereby enhancing cytotoxic T cell recognition [84].

The processing of Ii and regulation of CLIP removal constitute critical checkpoints in the MHC-II antigen presentation pathway. Dysregulation of these processes, through altered cathepsin activity or through enhanced CLIP retention, has emerged as a common mechanism of immune evasion in cancer.

## 5. Therapeutic Implications of CD74 in Cancer

### 5.1. Prognostic Associations

CD74 has emerged as a complex prognostic biomarker across many cancer types, reflecting its dual role in both immune activation and tumor cell survival. High CD74 expression can correlate with favorable or adverse outcomes depending on the biological context and TME.

In many malignancies, CD74 expression is linked to tumor progression and poor clinical outcome. Early studies showed that CD74 surface expression on malignant B cells not only marked tumor aggressiveness but also directly contributed to oncogenic signaling. Specifically, activation of CD74 in B cell chronic lymphocytic leukemia cells triggered a malignant cell survival cascade via NF-κB activation, suggesting that CD74 acts as a survival receptor enabling persistence of mature B cells after differentiation [36].

Recent research has further elucidated the prognostic landscape of CD74 across multiple tumor types. In pancreatic ductal adenocarcinoma (PDAC), CD74 expression is elevated, and it functions as a potential biomarker of disease progression. Mechanistic studies revealed that CD74 activation enhances proliferation, invasion, and inflammatory signaling via the TRAF6-NF-κB pathway. Knockdown of CD74 reduces PDAC cell growth both in vitro and in vivo, emphasizing its oncogenic and immunomodulatory roles [60]. Similarly, hepatocellular carcinoma (HCC) exhibits strong associations between CD74 expression, immune infiltration, and response to immunotherapy. High CD74 levels correlate with increased abundance of immune cell subsets within the TME, indicating that CD74 may act as a hallmark of immune engagement and a determinant of prognosis in HCC [61].

In melanoma, the MIF-CD74 axis has been implicated in both disease progression and therapeutic resistance. A recent review highlighted the importance of the MIF-CD74 pathway as a defining feature of melanoma biology and immune evasion [66]. In one study, CD74 was found to be a favorable prognostic marker associated with enhanced immune infiltration and improved survival in patients with stage IV melanoma [56]. Other studies showed that elevated MIF levels are associated with poorer prognosis, particularly in metastatic cases [85]. Dual inhibition of MIF and its homolog DDT has been proposed as a novel therapeutic strategy to overcome immune checkpoint inhibitor resistance in different types of cancer [86].

Beyond melanoma and HCC, CD74 has prognostic relevance in several other cancers. High CD74 expression predicts enhanced immunotherapy response in certain tumor types correlating with increased cytotoxic T cell infiltration and immune activation [87]. In cervical cancer, CD74 expression in tumor-associated macrophages (TAM) was shown to modulate response to therapy; CD74 blockade augmented the efficacy of neoadjuvant chemotherapy when combined with PD-1 checkpoint inhibition, implicating CD74 as a therapeutic co-target [73]. In clear cell renal cell carcinoma, CD74 expression was detected in both tumor cells and TAMs in over 90% of cases and functional studies have demonstrated that CD74 signaling enhances cancer cell proliferation and macrophage activation, although the precise crosstalk mechanisms remain to be clarified [88].

Finally, a recent pan-cancer analysis involving spatial transcriptomics confirmed that CD74 is significantly upregulated in most malignancies relative to normal tissue and serves as a predictor of overall prognosis [89]. In some cancer types, elevated CD74 expression was associated with signatures of M1 macrophage infiltration, reduced DNA repair gene expression, and enhanced immunogenicity in multiple tumor types. Interestingly, CD74 levels predicted improved chemotherapy response in breast cancer and were linked to potential drug-receptor interactions with novel small molecules such as HNHA and BRD-K55186349 [89]. Collectively, these studies underscore the multifaceted prognostic value of CD74, reflecting both tumor-intrinsic oncogenic signaling and its role as a modulator of tumor–immune interactions. CD74′s prognostic value varies by tumor type: favorable in immune-inflamed cancers such as melanoma and HCC, but adverse in malignancies driven by MIF-mediated survival signals such as lymphomas and PDAC.

### 5.2. Therapeutic Implications

#### 5.2.1. CD74-Targeted Therapies

Its restricted expression in normal tissues, combined with its rapid internalization upon ligand engagement, makes CD74 an attractive therapeutic target. One of the most extensively studied CD74-directed agents is milatuzumab (IMMU-115), a humanized IgG1κ monoclonal antibody that binds a cell surface epitope of CD74 [90]. Milatuzumab demonstrated potent antitumor activity in preclinical models and showed early signs of clinical efficacy in hematologic malignancies, particularly in B cell lymphomas and multiple myeloma, where CD74 is highly expressed [91,92,93,94]. Although initial results were encouraging (clinical trials NCT00603668 and NCT00989586), clinical progress ultimately stalled, highlighting the need for more effective targeting approaches and rational combination strategies.

To enhance therapeutic potency, milatuzumab was also evaluated as an antibody-drug conjugate (ADC) by linking it to doxorubicin, leveraging CD74-mediated internalization for intracellular drug delivery. This ADC exhibited strong antitumor activity in preclinical B cell tumor models; however, the clinical program in multiple myeloma was terminated in 2021 after insufficient efficacy was observed (NCT01101594). Despite these setbacks, milatuzumab has remained an important proof-of-concept molecule and has informed the design of next generation CD74-directed ADCs and combination regimens for leukemia, lymphoma, and other diseases.

#### 5.2.2. MIF Inhibitors for Cancer Therapy

In parallel with CD74-targeted cancer therapies, humanized monoclonal antibodies that neutralize MIF itself are also under development. One such agent, imalumab (BAX69) [95], was well tolerated and showed preliminary signs of antitumor activity in advanced solid tumors during a phase I clinical trial (NCT01765790) [96]. However, subsequent clinical development was discontinued following an overall benefit-risk evaluation (NCT02448810). Efforts have since shifted toward next generation MIF-blocking antibodies such as ON203 that exhibit improved biochemical and pharmacologic properties as compared to imalumab, and are emerging as promising immunotherapeutic candidates [97].

Beyond antibody-based strategies, small-molecule MIF inhibitors represent an additional approach to disrupt MIF-mediated tumor growth. Compounds such as ISO-1 inhibit CD74-dependent cancer cell survival signaling and render tumor cells more susceptible to chemotherapy or immunotherapy [98]. More recently, another small-molecule MIF inhibitor, CPSI-1306, was shown to suppress tumor growth in triple-negative breast cancer models by inducing tumor cell apoptosis by downregulating survival and proliferation pathways [99]. These findings suggest that pharmacologic MIF inhibition may offer a promising therapeutic strategy for limiting the progression and metastasis of cancer cells.

#### 5.2.3. CD74 in Immunotherapy and Combination Strategies

Given its intertwined relationship with immune activation and suppression, CD74 plays a crucial role in determining immunotherapy outcomes. Tumors co-expressing CD74 and MHC-II typically show higher infiltration by CD4^+^ and CD8^+^ T cells and exhibit improved responses to immune checkpoint blockade therapies such as anti-PD-1/PD-L1 therapies [61,87]. Conversely, in settings where CD74 signaling is dominated by MIF-mediated survival cues, this axis can limit immunotherapy efficacy. Consequently, dual blockade of MIF-CD74, in combination with checkpoint inhibitors, represents a promising avenue to overcome immunotherapy resistance and enhance T cell-mediated tumor rejection. Most recently, targeting TAM-derived CD74 in cervical cancer models was shown to potentiate immunotherapeutic responses [73]. Moreover, CD74 shapes the immune landscape by influencing macrophage polarization: high CD74 levels correlate with M1 macrophage infiltration, a phenotype linked to antitumor immunity and improved patient outcomes [89].

#### 5.2.4. CD74 in Cancer Vaccine Design

CD74 has attracted significant interest as a molecular tool in vaccine engineering for cancer immunotherapy. A key rationale for incorporating CD74 into vaccine constructs is that Ii naturally directs MHC-II complexes to endosomal and lysosomal compartments, where tumor antigens can be processed and loaded efficiently. By fusing tumor antigens to truncated or modified forms of Ii, researchers can force antigens into the MHC-II presentation pathway, substantially increasing the density and stability of peptide–MHC-II complexes on the surface of APCs [100,101,102]. This strategy enhances CD4^+^ T helper cell activation, which is crucial for sustaining effective CD8^+^ T cell cytotoxic responses and improving long-term antitumor immunity [103].

Modified Ii constructs also include replacing the “placeholder” CLIP peptide with tumor epitopes, and these tools dramatically increase MHC-II loading efficiency and augment CD4^+^ T cell responses in preclinical models [104,105]. This approach has demonstrated improved immunogenicity and superior tumor control as compared to non-Ii-linked vaccines. Improvement of personalized cancer vaccine design involving CLIP exchange and MHC-II epitope selection may be possible in the near future thanks to recent innovations such as the MHC2-SCALE platform, which enhances the identification of immunogenic neoantigens [106].

Collectively, these innovations highlight CD74′s value as a biotechnology tool for cancer vaccine optimization. By leveraging the natural trafficking of Ii and its role in peptide loading, vaccine constructs that incorporate Ii variants can enhance antigen presentation, amplify helper T cell responses, and improve the overall potency of antitumor immunization strategies.

## 6. Conclusions and Future Perspectives

CD74/Ii holds a uniquely multifaceted position at the intersection of antigen presentation, immune regulation, and cell signaling. Serving both as a chaperone and as a receptor, CD74 orchestrates multiple layers of immune visibility within tumors. Its roles in cancer underscore its value as both a therapeutic target and as a biomarker. Emerging research continues to reveal functions that extend far beyond its classical role in MHC-II trafficking, highlighting CD74 as a central modulator of tumor–immune interactions.

Therapeutically, CD74 is particularly attractive: its extracellular domain enables efficient targeting by antibodies and ADCs, while its intracellular signaling capacity offers opportunities to reprogram immune responses. As anti-CD74 antibodies, ADCs, and MIF pathway inhibitors progress through clinical development, and as integrative “omics” approaches better define its biomarker potential, CD74 is increasingly recognized as both a driver of tumor progression and a gateway to next-generation immunotherapies.

Future directions include clarifying how CD74 isoforms and post-translational modifications shape the MHC-II peptide repertoire in tumors, applying advanced immunopeptidomics to map these changes, and using therapeutic combinations such as CD74 blockade with interferon signaling activators or immune checkpoint inhibitors to suppress tumor growth. Altogether, acknowledging CD74 as a “master regulator” of antigen presentation reframes it from a passive scaffold to an active determinant of cancer immunity and therapeutic responsiveness.

## Data Availability

No new data were created or analyzed in this study. Data sharing is not applicable to this article.

## Abbreviations

The following abbreviations are used in this manuscript:

Ii	Invariant chain
MHC	Major Histocompatibility Complex
MIF	Macrophage Inhibitory Factor
CLIP	Class-II associated Invariant chain peptide
ER	Endoplasmic Reticulum
CIITA	Major Histocompatibility Complex class II Transactivator
ADC	Antibody-Drug Conjugates
DC	Dendritic Cell
APC	Antigen Presenting Cell
IL	Interleukin
VEGF	Vascular Endothelial Growth Factor
IFN	Interferon
CTSS	Cathepsin S
SPPL2	Signal Peptide Peptidase-Like 2
PDAC	Pancreatic ductal adenocarcinoma
HCC	Hepatocellular carcinoma
TME	Tumor Microenvironment
